# The Institutional Worker

**Published:** 1913-08-02

**Authors:** 


THE
### INSTITUTIONAL WORKER
**Being a Special Supplement to "The Hospital"**

### OUR BUREAU OF INFORMATION.

### Rules for Correspondents.

1. Every letter must be accompanied by the coupon to be cut from the back cover (inside page) of The Hospital, current issue, and must contain the name and address of the correspondent with pseudonym for publication if desired. Replies by post cannot be given, save under exceptional circumstances at the Editor's discretion.

2. Letters from Approved Homes in reply to special needs published in the Bureau should state terms and full particulars, and be sent prepaid under cover to the Editor of the Bureau with name written across coupon for identification.

3. Proprietors of Homes which have not yet been entered on the List of Approved Homes, but have spare accommodation likely to suit special needs, are invited to write for an application form for registration. The fee for registration, which includes two announcements of the Home in the Bureau and other privileges, is 10s.

4. All communications to be addressed to the Editor of The Hospital, 28 Southampton Street, Strand, London, W.C., and marked "Bureau of Information."

### INSTITUTIONAL FACTS AND FIGURES.

### The July Questions.

The answers to these questions appear to indicate that considerable interest is taken in the method of recording admission, etc., of patients, and in checking, sorting, analysing, and filing accounts for payment. We have only space this week to publish one selected answer to Question 1, and hope to publish other answers in the ensuing weeks.

### THE METHOD OF RECORDING ADMISSIONS.

Question 1 was as follows: State the character of your institution, and describe your system of recording the admission, treatment, and discharge of patients admitted to the wards. Give descriptions of the various forms in use for the purpose, the method of dealing with them in the process of registration, and the method of filing history and treatment sheets. Add your further remarks.

### Selected Answer.

This institution is an eye infirmary, containing 104 beds. Admission is by subscriber's "letter." Average annual number of in-patients, 1,600; average annual number of out-patients, 20,000. There are six surgeons between the infirmary, dispensary, and an additional outside dispensary. The following are the forms and books in use:

(1) A sheet of foolscap size, each of which bears the name of the surgeon on duty for the day, and on which are entered particulars of each new patient, viz.:

| | | |
|---|---|---|
| Name | Age | Occupation |
| Address | | |
| Employer | | |
| Recommended by Society | | |

The history and diagnosis of the case are then entered by the surgeon, after which the sheet is stamped with an official stamp "Admitted to House."

(2) An indoor admission book, the pages of which are divided up as follows:

Name

| Male | Female | Age | Surgeon | Disease | Admitted |
|---|---|---|---|---|---|
| | | | | | |

| Discharge | Result | | | Sheet No. |
|---|---|---|---|---|
| | Cured | Improved | I.S.Q. | |

And having an alphabetical index, the pages of which are again divided into "Male" and "Female" portions, so that names can be the more easily picked out. The necessary particulars are entered into this admission book.

(3) Two books, "The Employers' Book" and "The Societies' Book," exactly identical in ruling:

Name

| | | | | |
|---|---|---|---|---|
| Prudential | W. $\frac{2700}{1913}$ | Page 40 | | |

In column "Name" the name of the employer or society is entered; in the next column is entered the number of the sheet, preceded by the initial letter of the surgeon under whose charge the case is, to prevent confusion, thus: W. $\frac{2700}{1913}$ would represent that the sheet was number 2,700 of the year 1913 belonging to Dr. W.; in the next column is entered the number of the page of the admission book in which the case is entered.

(4) The "Residences of Patients" book is ruled thus:

| Town | 1 | 2 | 3 | 4 | 5 | 6...........31 | Total |
|---|---|---|---|---|---|---|---|
| | | | | | | | |

There being thirty-one small columns representing the days of the month to each page. At the end of each day the names of the towns from which patients have come (this applies to outdoor as well as indoor cases) are entered in the first column, and the number in the appropriate column according to the particular day of the month. The totals are extended and are thus easily available for the annual report statistics.

#### Procedure.

When the patient is sent to a ward the foolscap sheet is then firmly secured in a "cardboard book" fitted with a fixed pad of sheets of blotting-paper. The book is then hung at the top of the bed, beneath a small sliding wooden frame, in which is inserted a card, bearing in large type the name of the surgeon. The surgeon on his rounds enters all observations regarding his case on this foolscap sheet. Operations are denoted in a different colour of ink.

In (5), the operation book, are entered the name of the patient, the sheet number as before, and the nature of the patient; whilst in (6), the "classification of operations" book, a summary of the numbers of each different kind of operation is made on the same principle as adopted in the "residences of patients" book, and the totals from this book again form the basis of the statistics for the annual report.

When the patient comes to be discharged the house surgeon indicates on the sheet "C" for cured, "I" for improved, and "Q" for *in statu quo*. The sheets are then sent from the wards to the office, where the necessary particulars are entered in the remaining columns of the admission book, before described.

#### Method of Preserving Records.

The sheets are transferred to an Amberg numerical cabinet and filed, according to their number, in the cabinet allocated to each surgeon. When the patient after his discharge returns to the dispensary and produces his dispensary card (a small card of distinguishing colour for each surgeon, with all information as to hours and days of attendance, and bearing the same number as foolscap sheet) the sheet is easily found. The sheets of each year belonging to each surgeon after a space of three years, are bound in handy volumes containing an alphabetical index of the names of the patients.

#### General Remarks.

The combination in one book of admission and discharge saves wearisome repetition of writing patients' names, etc., apart from presenting at sight the dates of a patient's admission and discharge and result. This is very essential in our case where in a commercial and industrial area the number of certificates to be given to patients for their employers and societies is great. The employers' and societies' books are very useful to this extent, that employers, before making allocations, usually wish to be acquainted with the number of men admitted from their works and average number of days spent by each, and the same information is desirable to have ready in concentrated form regarding the members of the various societies.—"Sonia."

### QUESTIONS for AUGUST.

August is a holiday-making time, and therefore the questions we are publishing for this month are of such a nature as to be easy to answer without recourse to reference books. Question 2 touches on a branch of philanthropy which is coming greatly to the fore, and practical workers in this field are invited to give their experience which cannot fail to be of considerable help to the many beginners whom this work is attracting. Question 1 should appeal to the secretariat of any institution, and their views, with the reasons for such views, will be welcome.

**Question 1.**

**Is it or is it not an advantage for a hospital to possess an addressograph? Give reasons, and if an advantage give particulars of cost.**

**Question 2.**

**Give a week's menu for dinners for mothers in connection with Schools for Mothers, with the daily cost for 20 people, bearing in mind the need for nourishing food and for strict economy.**

RULES.

The following rules must be observed:—

1. Contributions must be written on one side of the paper. They must bear the name and address of the sender and be accompanied by coupon to be cut from the back cover (inside page) of the current issue of The Hospital. A pseudonym must be chosen if the name is not to be published.
2. They must be addressed to the Editor of The Hospital, 28 & 29 Southampton Street, Strand, London, W.C., so as to reach him by the last day of the month for which the questions are set, and must be marked in the left-hand corner "Facts and Figures."

**A minimum payment of Five Shillings will be made for each published answer.**

### *THE SICK AND IN NEED.*

### A Very Urgent Need.

We have heard from the lady who has interested herself in this case that the wife of the man with phthisis has obtained a post which will enable her and her children to exist during the man's absence.

### Home Wanted for District Nurse.

The case you write about of a district nurse only a little over 30, who has completely broken down in health with bad heart disease is a sad one. You say she has been told she cannot do any more nursing work, and her only other accomplishment is needlework. She is absolutely without means, and if a free home cannot be found for her she will have to go to a workhouse. We should like some further details to enable us to advise or suggest a course. Is she a Queen's Nurse, or has she worked for any public nursing association? Is she a member of the Royal National Pension Fund? Is not she drawing sickness benefit under the National Insurance Act? Is she able to get about and help herself?—V. H. B.

### THE EDITOR'S LETTER-BOX.

### *EMPLOYMENT AND TRAINING.*

### Nursing Home in France.

A trained nurse is anxious to hear of a place on the Continent, preferably in France, where there is an opening for a nursing home. She would be joined by her sister, also a trained nurse, who has two years' training in massage, electricity, and medical gymnastics, and between them they have medical, surgical, midwifery (C.M.B.), and fever training. Both sisters speak German, French, and a little Italian. Anyone knowing a place where a nursing home is really a need are asked to write to "Sirod," 169q.

### Post in Chinese Mission Hospital.

"Guy's Hospital Gazette" states that the hospital at the treaty port of Wenchow, in the province of Chekiang, which is in connection with a mission, has a vacancy for a recently qualified man who would have the opportunity of gaining experience in many diseases not seen in this country, and learning a little at first hand of China and the Chinese. Surgical work is to be had in abundance. Further particulars may be obtained from Dr. W. E. Plummer, 24 Granville Road, Sidcup, Kent.

### Training at Eighteen.

The Alexandra Hospital for Children with Hip Disease receives probationers at 18, as does also the Home and Infirmary for Sick Children, Lower Sydenham. You should get "How to Become a Nurse," and read what is said in the first chapter in this connection.—M. G.

### *ACKNOWLEDGMENTS.*

**"Sirod."**—We have put your inquiry in our columns. We are advised there is no English nursing home in Pau, and that there is a large demand during the season (November to May) for trained nurses. The British Vice-Consul at Pau, H. T. H. Hewetson, Esq., would probably be able to answer any specific questions you sent him.

**M. E. F.** — We are very glad to know the case is now provided for.

**J. W.**—You will note that the case has now been provided for. We are asked to say that your suggestion may be of use in the future.

**H. M. B.**—Thanks for letter and enclosure; we already had the matter in hand.

**E. A.**—Please read our rules. We do not give postal replies. You should consult the Colonial Intelligence League, 36 Tavistock Square, W.C.; or the British Women's Emigration Society, Imperial Institute, S.W.

*Letters with enclosures have been received and attended to from M. Pike.*

*Letters and postcards have been received and attended to from: Dr. Norton, Mr. G. Sillem, Mrs. Barker, Miss Ada Parr, Nurse Holland, Miss M. Buck, Mr. F. H. Newton, Miss R. Sharp.*

### *MISCELLANEOUS.*

### The Flag for a Hospital to Fly.

In reply to your question what is the proper and correct flag to fly upon a hospital, we would refer you to the article in The Hospital of June 17, 1911, p. 293, where the whole question as it affects England is thoroughly gone into. In a previous issue of The Hospital—that for June 10, 1911, p. 263, this right as it affects hospitals abroad is dealt with. As regards England, as the former article states, the hospital flag to fly in this country is the Union Jack. This is, however, merely a matter of courtesy; a voluntary hospital has no "right" to fly the Union Jack. It may fly the municipal flag if it is associated with the borough in any corporate capacity, and it may also fly a special flag if it choose to have one. In time of war, when within the zone of operations, it must fly the Red Cross flag in addition to the national ensign if it is a public hospital. We would recommend you to read the articles referred to.—W. H. C.

### Consult Your Panel Doctor.

We do not advise you to insert in this paper or any other an advertisement of the kind you suggest. The long duration of your trouble and the fact that you have obtained only temporary relief from the doctors you have consulted are certainly enough to make you feel despondent, and you have our sympathy. We feel quite sure from what you tell us that you are being frequently re-infected. You must have been really cured many times, but have caught fresh infections. How you manage to do this we cannot say, because we do not know your occupation, your habits, and the conditions in which you live. Your case requires not so much curative medicine (though that, of course, is important too), as prevention of further attacks. We are quite convinced there must be some source from which you acquire these fresh infections; and we suggest that you should go to your panel doctor and invite him to attempt to find out what and where that source is. If he is unable to do so, ask him to recommend you to the hospital: a week in the ward would no doubt cure you, and if you afterwards relapsed it would be clear that you were being somehow re-infected. It is just conceivable, though not very likely, that the trouble resides in the appendix; but this is a point upon which your doctors can advise you far better than we can.—H. C., Carlisle.

**The Editor is prepared to make known without charge the needs of any who may be sick or in difficulties, and to guide them in making choice of Homes for treatment or convalescence. Reduced terms can often be arranged with Homes on the Approved List for those unable to pay full fees. No Home is included in the Approved List without medical and other testimony to its good management. Queries are specially invited from those who are engaged in any kind of philanthropical work.**

### Editor's Notices.

**Contributions :**

Contributions should be written, or preferably typed, on one side of the paper only, and all articles sent in are accepted upon the distinct understanding that they are forwarded to THE HOSPITAL only.

The Editor cannot undertake to return MSS. not used, but when a stamped directed envelope is enclosed the MSS. may be returned if a special request is made.

Accepted articles and paragraphs of news will be paid for after publication at the scale rate.

**Address :**

To prevent delay all contributions and letters on editorial business must be addressed exclusively to the Editor, "The Hospital," 29 Southampton Street, Strand, London, W.C. It is important that this regulation shall be strictly observed.

**Correspondence :**

Correspondence on all subjects is invited. The name and address of correspondents must be given as a guarantee of good faith, but not necessarily for publication.

**Special Articles :**

Special articles are invited, and questions, inquiries, and paragraphs upon all matters relating to the work, administration and management of general, special, mental, fever, cottage and convalescent hospitals, sanatoria, homes, institutions, societies and organisations for the treatment or care of the sick, injured and dependants of all classes. Every matter affecting the interests and welfare of the staff of all grades working in these institutions will receive special consideration.

**Administrative Medicine, Research and Items of News:**

Special terms will be paid for approved articles on subjects in this wide field by experts who have made some section of it their special study and interest. We wish to give prominence to every movement tending to advance accuracy of diagnosis, efficiency in treatment, and the development of modern methods to eradicate disease and promote the welfare of the suffering.

**A Bureau of Information :**

We invite inquiries and applications for information and help in providing for that numerous class of sufferers who require special care or house-room which they cannot obtain in their own homes or secure for themselves. We cannot, however, prescribe, or recommend practitioners.

**Books for Review :**

Publishers are particularly requested to send advance proofs of any new books of importance whenever possible, as the Editor has made arrangements to publish immediate reviews on a new plan.

**Photographs, Plans, Blocks, and Illustrations :**

It is requested that wherever possible MSS. may be accompanied by illustrations in any of the above forms. The name of the sender and of the article to which it belongs should be written on the back of each photograph, drawing, or block for purposes of identification.

**Local Papers :**

Newspapers containing reports or news paragraphs should be marked and addressed to the Sub-Editor.

**The Coupon System :**

A coupon will be found at the bottom of the third inside page of the cover each week. This coupon must be attached to every question or inquiry to which an answer is desired in THE HOSPITAL.

### Are You a Subscriber ?

THE ever-increasing circulation of THE HOSPITAL often renders it a matter of some difficulty to procure a copy of the current issue at the local newsagent's. By sending a subscription to the Journal either through a bookseller or direct to the Publishing Offices you guard against the contingency of THE HOSPITAL being sold, and ensure a copy reaching you almost immediately upon publication.

When seeking a post this is a great help, since it enables you to send in your application without delay, an advantage which may secure you the appointment. In addition to this, as a subscriber, no matter how many times you may change your address, or in what part of the country you may be at the moment, a postcard will ensure that you receive THE HOSPITAL on the same day and at the same hour as when at home.

**Subscriptions** may begin at any time, and are payable in advance. Cheques and Post-Office Orders should be crossed London County and Westminster Bank, Covent Garden Branch, and made payable to the Manager of THE HOSPITAL.

RATES OF SUBSCRIPTION (payable in advance).

| | | |
|---|---|---|
| UNITED KINGDOM. | | |
| Three Months | (including Postage) ... ... ... ... | 2s. 0d. |
| Six Months | do. ... ... ... ... | 4s. 0d. |
| Twelve Months | do. ... ... ... ... | 6s. 6d. |
| FOREIGN AND COLONIAL. | | |
| Three Months | (including Postage) ... ... ... ... | 2s. 6d. |
| Six Months | do. ... ... ... ... | 5s. 0d. |
| Twelve Months | do. ... ... ... ... | 8s. 8d. |

*SUBSCRIPTION FORM.*

*Please send me* THE HOSPITAL *for..............months, commencing with your issue of.............................., for which please find enclosed............................*

*Name*..................................................

*Address* ..................................................

..................................................

*Date*....................................

*To* .................................... *Newsagent.*

### Manager's Notices.

**Letters relating to the publication, sale, and advertising departments of "The Hospital" must be addressed to The Manager, "The Hospital," The Hospital Building, 28 and 29 Southampton Street, London, W.C.**

**Advertisements :**

To ensure insertion, all advertisements for THE HOSPITAL must reach the Manager not later than Wednesday morning in each week. For scale of charges see pages vii and 4.

**Remittances:**

**It is especially requested that remittances be made by Cheque or Postal Order, and in halfpenny stamps only when the amount is under sixpence.**

### HANDBOOKS
### For TRAINED WORKERS

POCKET·GUIDE SP SERIES

**1/- net. 1/1 Post free.**

**Bound in Limp Cloth.**

**Suitable Size for the Pocket.**

**The works which comprise the S.P. Pocket Guide Series are intended for ready reference, consequently the aim of the Publishers has been to make them as concise and lucid as possible. Each book is written by an expert on the subject of which it treats, and the utmost reliance therefore can be placed in the information it imparts.**

**Essentials of Fever Nursing.** By LYTTON MAITLAND, M.D. (Lond.), M.B., B.S., D.P.H. (Camb.).

**Manual and Atlas of Swedish Exercises.** (*With over 60 Illustrations.*) By THOMAS D. LUKE, M.D., F.R.C.S.

**Bandaging Made Easy.** (*Illustrated with over 90 Diagrams.*) By M. HOSKING, Sister-in-Charge, Tredegar House, Bow, E.

**How to Write and Read Prescriptions.** By LYTTON MAITLAND, M.D. (Lond.), M.B., B.S., D.P.H. (Camb.).

**Principal Drugs and their Uses.** By A PHARMACIST.

**Asepsis and How to Secure It.** By H. W. CARSON, F.R.C.S.

**Treatment after Operations.** *Rules for Nursing after General and Special Operations.* By MARY WILES.

**The Nurse's Duties before and during Operations.** By MARGARET FOX, Matron, Prince of Wales's General Hospital, Tottenham.

**Other works, in amplification of this Series, will be announced in due course.**

*Published by*

**The SCIENTIFIC PRESS, Ltd.**

**28/29 Southampton Street, STRAND, LONDON, W.C.**

